# Data Sharing: A New Editorial Initiative of the International Committee of Medical Journal Editors. Implications for the Editors´ Network

**DOI:** 10.5935/abc.20170054

**Published:** 2017-05

**Authors:** Fernando Alfonso, Karlen Adamyan, Jean-Yves Artigou, Michael Aschermann, Michael Boehm, Alfonso Buendia, Pao-Hsien Chu, Ariel Cohen, Livio Dei Cas, Mirza Dilic, Anton Doubell, Dario Echeverri, Nuray Enç, Ignacio Ferreira-González, Krzysztof J. Filipiak, Andreas Flammer, Eckart Fleck, Plamen Gatzov, Carmen Ginghina, Lino Goncalves, Habib Haouala, Mahmoud Hassanein, Gerd Heusch, Kurt Huber, Ivan Hulín, Mario Ivanusa, Rungroj Krittayaphong, Chu-Pak Lau, Germanas Marinskis, François Mach, Luiz Felipe Moreira, Tuomo Nieminen, Latifa Oukerraj, Stefan Perings, Luc Pierard, Tatjana Potpara, Walter Reyes-Caorsi, Se-Joong Rim, Olaf Rødevand, Georges Saade, Mikael Sander, Evgeny Shlyakhto, Bilgin Timuralp, Dimitris Tousoulis, Dilek Ural, J. J. Piek, Albert Varga, Thomas F. Lüscher

**Affiliations:** 1 Editors´Network; 2 Armenian Journal of Cardiology; 3 Archives des maladies du cœsur et des vaisseaux Pratique; 4 Cor et Vasa; 5 Clinical Research in Cardiology; 6 Archivos de Cardiologia de Mexico; 7 Acta Cardiologica Sinica; 8 Archives of Cardiovascular Diseases; 9 Journal of Cardiovascular Medicine; 10 Medicinski Zurnal; 11 SAHeart; 12 Revista Colombiana de Cardiologia; 13 Kardiyovaskuler Hemsirelik Dergisi; 14 Revista Española de Cardiología; 15 Kardiologia Polska; 16 Cardiovascular Medecine; 17 Cardio News; 18 Bulgarian Journal of Cardiology; 19 Romanian Journal of Cardiology; 20 Revista Portuguesa de Cardiologia; 21 Cardiologie Tunisienne; 22 The Egyptian Heart Journal; 23 Basic Research in Cardiology; 24 Austrain Journal fo Cardiology; 25 Cardiology Letters; 26 Cardiologia Croatica; 27 Thai Heart Journal; 28 Journal of the Hong Kong Colleage of Cardiology; 29 Seminars in Cardiovascular Medicine; 30 Cardiovascular Medecine; 31 Arquivos Brasileiros de Cardiologia; 32 Sydänääni (Heart Beat); 33 La Revue Marocaine de Cardiologie; 34 Der Kardiologie; 35 Acta Cardiologica; 36 Heart and Blood Vessels; 37 Revista Uruguaya de Cardiologia; 38 Korean Circulation Journal; 39 Hjerteforum; 40 Heart News; 41 Cardiologisk Forum; 42 Russian Journal of Cardiology; 43 Anatolian Journal of Cardiology; 44 Hellenic Journal of Cardiology; 45 Archives of the Turkish Society of Cardiology; 46 Netherlands Heart Journal; 47 Cardiologia Hungarica; 48 European Heart Journal

**Keywords:** Editorial Ethics, Scientific Process, Data Sharing, Clinical Trial, Trial Registration, Authorship, Conflict of Interest, Big-data, Scientific Journals

## Abstract

The International Committee of Medical Journal Editors (ICMJE) provides recommendations to improve the editorial standards and scientific quality of biomedical journals. These recommendations range from uniform technical requirements to more complex and elusive editorial issues including ethical aspects of the scientific process. Recently, registration of clinical trials, conflicts of interest disclosure, and new criteria for authorship - emphasizing the importance of responsibility and accountability-, have been proposed. Last year, a new editorial initiative to foster sharing of clinical trial data was launched. This review discusses this novel initiative with the aim of increasing awareness among readers, investigators, authors and editors belonging to the Editors´ Network of the European Society of Cardiology.

The Editors´ Network of the European Society of Cardiology (ESC) is committed to promoting the implementation of high-quality editorial standards among ESC National Societies Cardiovascular Journals (NSCJ).^1-4^ NSCJ play a major role in disseminating high-quality scientific research. However, they also play a relevant role in education and harmonization of clinical practice.^3^ Most NSCJ are published in local languages, but many have English editions and have gained international scientific recognition.^1-4^ NSCJ well complements official ESC journals and, altogether, provide an effective means to disseminate European cardiovascular research. In a globalized and highly competitive editorial environment, promoting high quality editorial standards remains of paramount importance to increase the scientific prestige of NSCJ.^1-4^ From its conception, the Editors´ Network strongly advocated for the adherence to the uniform recommendations of the International Committee of Medical Journal Editors (ICMJE).^1^ In its mission statement document the Editors´ Network committed to adapt NSCJ to follow these general editorial recommendations.^1^ However, NSCJ are highly heterogeneous in scope and contents and these new recommendations should be embraced progressively, considering currently existing editorial policies and the editorial freedom of the NSCJ.^1-4^

Ethical issues play a growing role in ensuring the credibility of the scientific process.^5-13^ Biomedical research relies on trust. However, transparency also represents a major tenet in the scientific process.^5-8^ This review will discuss the new editorial recommendations on data sharing issued by the ICMJE.^14^ Novel ICMJE recommendations always appear as provocative, and often as too ambitious, when initially presented. Moreover, implementation of editorial changes is rather demanding from a technical and logistical viewpoint. Adherence to novel editorial initiatives is challenging not only for editors, but also for the entire scientific community. Therefore, many Editors have a natural tendency to avoid stepping ahead as early adopters of new editorial experiments and usually prefer to keep moving within their comfort zone until the sea change has matured.^1-4^ However, experience has taught us that all editorial initiatives developed by the ICMJE eventually prevailed and played a critical role in maintaining the credibility of the scientific process.^9-13^ Highly successful recent examples include trial registration, a conflicts of interest initiative and the new requirements for authorship.^9-13^

The novel ICMJE recommendations on data sharing^14^ are discussed herein from a didactic perspective with the aim to provide new editorial insights and, hopefully, to be progressively adopted and implemented by the NSCJ.

## Sharing clinical trial data: the new ICMJE proposal

The ICMJE considers that there is a moral obligation to responsibly share the data generated by clinical trials.^14^ The rationale underlying this global endeavor is that patients have assumed a risk by accepting to participate in a trial. Accordingly, making the obtained data publicly available represents a responsible initiative to facilitate the advancement of science. Sharing the data would increase trust in the conclusions reached by trials. Indeed, data sharing allows confirmation of the results by independent research.^14^ Furthermore, new hypotheses may be pursued by different groups of investigators. This initiative may foster the leveraging of data to answer different research questions not contemplated in the original study. If science becomes an open process, then many researchers would benefit by taking advantage of reliable data generated somewhere else. Therefore, data sharing emerges as the best way to ensure that all the information gathered by trials is made freely and widely available, so that it can be readily used to advance scientific knowledge.^14^ The use of previously collected data to further advance science is difficult to criticize. As discussed, this honours the volunteerism of the patients who signed up and consented to participate in a trial.

Governments, funding agencies, scientific societies, the industry and even the lay society growingly demand sharing clinical trial data. Therefore, the ICMJE suggests that editors should help to meet this ethical obligation by devising new editorial policies specifically addressing this issue.^14^ Proponents of open science should be pleased by this new editorial requirement of sharing clinical trial data.^14^

The first consideration is to clarify what a clinical trial is exactly. According to the ICMJE definition, a clinical trial is a study that prospectively assigns people to an intervention in order to assess the cause-and-effect relationship between that intervention and the ensuing health outcome.^5^

The ICMJE considers that sharing *de-identified* individual patient data should become part of the publication process of clinical trials.^14^ This strategy protects patient´s confidentiality rights. The requirement, however, is restricted to the individual-patient data underpinning the results presented in the published article. Importantly, a clear plan for data sharing should be disclosed at the time of initial trial registration and should be also presented at the time of manuscript submission. The proposal requires clinical trialists to declare that they will share their data publically as a prerequisite for publishing the trial.^14^ They should promise to freely release individual patient raw data at the time they submit the manuscript for consideration.

It is important to keep in mind that clinical trial registration was a previous ICMJE editorial initiative aimed to address problems related to publication bias (selective publication of positive trials), endpoints inconsistency and redundant research.^9,10^ Potentially, public repositories provide an optimal tool not only for initial trial registration but also for individual-patient data sharing. From now on the plan for data-sharing would be an important step of the clinical trial registration initiative.^9,10,14^ Details on whether the data would be freely available upon request, or only after a formal application that eventually will be approved after an agreement is reached on data use conditions, should be presented. Finally, it has been proposed that the data should be made public no more than 6 months after publication of the original study in the journal.^9,10,14^ *Clinicaltrials.com*, a widely used non-for profit scientific repository,^9,10^ has already adapted its registration platform to specifically clarify data-sharing plans at the time of clinical trial registration.

Obviously, this editorial initiative may have profound consequences on the planning, conduction and reporting of clinical trials and, in fact, may deeply influence research and publication strategies.^14^ As a result, the idea is to implement this requirement for any clinical trial that begins to enroll patients 1 year after the official adoption of this editorial policy by the corresponding journal.^14^ The initiative will also have major implications for the editorial process. Indeed, Editors are supposed to monitor the data sharing process and, eventually, address potential irregularities. These might include requests of clarification to the authors, notification to academic institutions, publication of expressions of concern or even retractions.

Finally, the ICJME acknowledges that the rights of the investigators and sponsors should be protected.^14^ Moreover, credit to the original report should be granted by including a unique identifier of the data set. It is emphasized that credit should be always given to the original investigators that posted the data after publication of their research. Furthermore, additional investigators using these databases should request collaboration of the investigators that originally collected the data to ensure adequate data interpretation, management and analysis.

## Challenges of data sharing

Although it appears clear that this initiative will further improve transparency and the overall integrity of the scientific literature, some remaining issues need to be addressed. There is inherent resistance to embrace open science initiatives from some academic institutions or investigators that defend the idea of exploiting their own data.^15,16^ Until now clinical researchers were discouraged from working with clinical trial data they did not generate themselves.^15,16^ Likewise, trialists tended to see trial data as their personal property and would routinely refuse requests for data sharing. In fact, until very recently most researchers and pharmaceutical industry groups were opposed to making raw data available after trial publication. This practice, however, differs from other disciplines (as genomics or economics) where data sharing has been common place for a long time.^15,16^

Obtaining reliable, high-quality original data requires a major research effort. Allowing a sufficient period of time from the time of article publication to the need to share the raw data would give original investigators the possibility of publishing additional subgroup analyses from their own data.^14^ This new proposal will further increase the pressure on academic investigators that frequently do not have the required resources to publish their subsequent analyses and require time to prepare the new manuscripts.^14^ Notably, most researchers have no experience with the process of releasing or dealing with public data. Furthermore, the effort and resources required to organize the raw data in a way that would be comprehensible to other investigators remain a cause of major concern.^14^ This would require technical support and adequate funding.

Data-access to non-trial researchers may disclose problems not recognized by the initial investigators. Although this will increase transparency and, therefore, trust in trial results, it might also generate confusion and undue scientific controversies. It is difficult to envision how the new researchers will gain the required detailed knowledge of the complicated datasets enjoyed by the original trial investigators.^14^ A reliable assessment of the data requires a deep knowledge on the study background and to be able to properly address many nuances and practical considerations. These include precise information on the way variables were defined, how data was collected and how results were finally coded and entered into the database. The initiative might be fraught with problems related to incorrect analysis resulting in inaccurate results and erroneous interpretations, potentially damaging science.^14^

Finally, Editors, already deluged with work, will need to check that all of the raw data of the published articles eventually has been released as promised. Different results may emerge from misconceptions regarding what data should be analysed to answer specific questions.^14^ If there are differences in results, it will be difficult to decide which analysis provides the most accurate reflection of the data. This could generate undue *scientific noise*, with contradictory results and rectifications, which may generate confusion and frustration in the scientific community. Finally, this may also promote the simultaneous publication in several journals of conflicting results from the same database by different groups.^14^

As many issues still should be clarified, the ICMJE asked for feedback on its preliminary editorial proposal on clinical trial data sharing.^14^ Obviously, the initiative will only gain the required maturity from the experience gained during its adoption and implementation.

## Previous initiatives on data sharing

Several leading academic entities previously have worked in this field. The *British Medical Journal* pioneered an editorial initiative of data sharing.^17^ In 2012 this policy took effect only for trials on drugs and devices but, in 2015, the requirement of data sharing on request was extended to all submitted clinical trials.^17^ It has been proposed that individual patient data may also be of major value during the *peer review* process by permitting independent verification of the results before final publication.^18^ Although this initiative might be of potential value most reviewers are already deluged with work and this extra task could generate fatigue and burn out phenomena. In addition, many good clinical reviewers do not have the expertise required to manage data and to perform confirmatory statistical analyses.^18^ Some journals, as JAMA, previously developed some related editorial initiatives including the request for independent statistical analyses by an academic statistician of industry-sponsored trials.^19^

The World Health Organization (WHO) and the Institute of Medicine (IOM) previously made important declarations on clinical trial transparency. In this regard, the IOM issued specific guidelines for trial data sharing.^20^ WHO initially presented a statement on public disclosure of clinical trial results and, subsequently, encouraged sharing of research datasets whenever appropriate.^21-23^ More recently, the WHO developed global norms for sharing data and results during public health emergencies, with special focus on clinical, epidemiologic, and genetic features of new infectious diseases and experimental therapeutics and vaccines. In emergency situations, data needs to be shared quickly before the information is formally published.^23^

Finally, the National Heart, Lung and Blood Institute (NHLBI) presented detailed data-sharing practices allowing public access to trial raw data and developed a data repository currently including over half a million patients from over 100 trials and observational studies.^24^ In 2015 the NHLBI discussed its intent to make public the digital data from its funded trials.^24^

## Platforms and repositories

Up to 30,000 clinical trials are conducted annually worldwide generating a huge volume of patient-level raw data.^25^ Currently, however, available portals for data sharing are still not adequate. Most of them require a time consuming request, including a detailed research proposal with the study design, main endpoints and a statistical plan.^25^ The submitted proposal is then reviewed by an independent research panel that decides whether to approve the request for data.^21,25,26^ Currently, this process takes too long and when eventually the data is obtained oftentimes it is not readily usable.^25^ However, the means to facilitate data sharing from the data holder to the researcher may be cumbersome and challenging to implement. Some systems provide an electronic form or template.^21^ Nevertheless, when these are not available, a *de novo* proposal should be generated outlining the purpose, the statistical analysis plan, the research team, and potential conflicts of interest. The review process may come from an internal or external review panel selected by the data holder or by a third party.^25-27^ Finally, data can be shared through a public website or by direct communication between the data holder and the researcher. In most cases, however, controlled access is required. Before any analysis is started, reviewing all the accompanying documentation to assist the researcher in the understanding of the original clinical trial and the methodology used remains critical. Furthermore, the data holder may require a legally binding data sharing agreement and should be available to provide the required support should questions arise.^27^

Major care should be taken to prevent the perils that may undermine the value of data sharing.^14^ Data from trials should be responsibly used.^28^ A recent survey from UK Clinical Trial Units disclosed some potential risks associated with data sharing.^29^ These basically included a) misuse of data, b) incorrect secondary analyses, c) resource requirements and d) identification of patients.^29,30^ Researchers are responsible for presenting the data in a format amenable for external secondary use. Repositories should be prepared to make raw data available in standardized platforms in a fully comprehensive manner. Data sharing from trials with anonymized patient-level data with associated metadata and supporting information should be made available to other researchers following an independent analysis of the research proposals. Developing and adopting standard approaches to protecting patient privacy are urgently required.^14^ Finally, an adequate infrastructure should be organized to support effective data sharing. In this regard, the role of the industry is significantly growing as demonstrated by some joint initiatives, such as the Yale University Open Data (YODA) project.^16,31^

Some academic research organization consortiums particularly focussed on the study of cardiovascular diseases,^32^ have developed interesting tools for data sharing. This cardiovascular initiative requires presentation of a standardized request in a Web portal. Proposals are to be analyzed by a scientific committee, including members designated by the consortium and a statistician along with the trial's principal investigator. The idea is to ensure an adequate use of the data base and correct statistical analyses, while averting the problem of multiple investigators proposing the same analyses.^32^

## Statistical issues

Statisticians play a key role in developing data sharing strategies.^19^ They should be involved from the very beginning to organize the research strategy and the required analytical techniques.^19^ In this scenario statisticians should move from their classical role as data gate-keepers to that of data facilitators.^19^ A data sharing working group of medical research statisticians has been recently created from the pharmaceutical and biotechnological industry and from academia. The idea was to address the technical and statistical challenges of accessing research data for re-analyses. Specific techniques are required to ensure adequate data manipulation to convert the data initially collected and entered in the data base into data that is analytically usable. Converting raw data into standardized formats may be challenging. Moreover, familiarity with the required statistical programing language is necessary. Independent statisticians should play a major role in guiding the principles of re-analysis based on the researchers´ request while, at the same time, guarding against misleading conclusions. They should be fully aware that additional analysis may yield different results compared with the original analyses. Accordingly, they should be prepared to face criticism but, at the same time, they should be able to openly challenge previous statistical methods.^19^

Statistical guidance may be required for appropriate interpretation of results from re-analyses where different methods have been utilized. In particular, it is important to keep in mind the inherent risk of over-interpretation of the results from multiple subgroup analyses.^33^ Likewise, documents for best practices in data anonymization have been developed.^34^ Statisticians should be also familiar with this methodology. Risk to patient privacy can be mitigated by data reduction techniques. Data holders are responsible for generating de-identified datasets to offer protection for patient privacy through masking or generalization of main identifiers. In addition, legally binding data sharing agreements should include a compromise not to attempt to identify patients.^34^ In particular, it is recommended that data use agreements are signed by the data holder and researchers. Only appropriately qualified named researchers should be granted access to the data. Finally, high security levels should be implemented for data transferring. Resources, costs and effort required to make patient-level data available for third party research may be considerable and, therefore, adequate funding should be organized.^34^

## Credit to the original authors

A clear motivation for researchers to conduct randomized clinical trials is the opportunity to publish different studies in addition to the main manuscript with the primary endpoint. These secondary analyses may be of major value to unravel new findings from the original dataset.^35,36^ Many have proposed that the time to open the process of data sharing should be extended to 2 years, or even to 5 years in selected complex or large studies. This will allow a precious time for original investigators to further scrutinize and analyze in depth their own data. As blinding is necessary during trial execution, once the study is completed the research teams concentrate on publishing the primary findings as soon as possible. Following this, usually there is a series of pre-planned additional analyses. These studies are organized by collaborative research teams from different institutions, but usually with relatively poor support. Secondary analyses are also very important for co-investigators and junior scientists. To respect this legitimate interest an extension from the 6 month-period after the primary data has been published has been advocated.^35,36^

Academia rewards scientists with recognition for making their discoveries public. Credit should be granted to the original researchers that create data sets that other investigators find useful.^14,15^ Otherwise, original investigators may be tempted to consider research parasites those performing secondary analyses of their data. Furthermore, mechanisms are required to ensure that the external analyses are conducted adequately and not merely to undermine the original findings. Direct collaboration between primary and secondary researchers is, therefore, necessary to ensure proper data analysis and interpretation.^14,15^ The original investigators that designed and conducted the trial and obtained sources of founding deserve to receive the adequate scientific credit.^28^

## Conclusions

The data transparency revolution is here to stay. This is just another step ahead into a culture of open science and it is clear that we are at the dawn of a new age.^37,38^ Several European National Societies have already developed registry programs in which the registries databases are public for the use of their members.^39^ Major challenges and hurdles in the adoption and implementation of the new ICMJE recommendation should still be overcome.^40^ Experience gained by leading journals will eventually allow a balanced compromise between the interests of the original researchers and that of the scientific community as a whole. NSCJ should progressively adapt their policies to increase awareness of the importance of data sharing and promote policies designed to enhance transparency in biomedical research.
